# A narrative review on artificial intelligence in neurosurgery: ethical challenges and implementation considerations

**DOI:** 10.1097/MS9.0000000000004246

**Published:** 2025-10-30

**Authors:** Tirath Patel, Hamza Yousuf Ibrahim, Fathimathul Henna, Fatima Nasir, Abbas Hussain, Rahma Naveed, Aziz Ur Rehman, Syeda Ramish Zehra Kazmi, Bhumi Daishik Patel, Nikhilesh Anand, Richard M. Millis

**Affiliations:** a Department of Neurosurgery, Trinity Medical Sciences University School of Medicine, Kingstown, Saint Vincent and the Grenadines; b Department of Surgery, Jinnah Medical and Dental College, Karachi, Pakistan; c Department of Surgery, Dubai Medical College for Girls, Dubai, United Arab Emirates; d Department of Surgery, Akhtar Saeed Medical and Dental College, Lahore, Pakistan; e Department of Surgery, Liaquat National Hospital and Medical College, Karachi, Sindh, Pakistan; f Department of Surgery, Windsor University School of Medicine, Cayon, Saint Kitts and Nevis; g Department of Medical Education, University of Texas Rio Grande Valley, Edinburg, Texas, United States of America; h Department of Pathophysiology, American University of Antigua, Saint John, Antigua and Barbuda

**Keywords:** artificial intelligence, ethics, implementation, neurosurgery

## Abstract

**Introduction:**

Artificial intelligence (AI) is revolutionizing neurosurgery by enhancing diagnostic precision, surgical planning, and postoperative management. However, its integration raises ethical concerns related to bias, privacy, accountability, and the potential dehumanization of healthcare. This review focuses on navigating these challenges while maximizing AI’s potential in improving patient care.

**Methodology:**

A narrative review was conducted by identifying studies from PubMed, Cochrane Library, and Google Scholar databases. The search utilized the following keywords: “artificial intelligence,” “neurosurgery,” “machine learning,” “data privacy,” “robotic surgery,” “ethics,” and “bias.” The review primarily focused on issues of dataset bias, data privacy, and the need for transparency and accountability in clinical decision-making.

**Results and critical insights:**

AI significantly improves diagnostic accuracy and the management of neurological conditions; however, it also poses risks, such as exacerbating healthcare disparities and compromising patient data security. Recommended strategies include the development of ethical frameworks, inclusion of diverse datasets, and fostering surgeon–AI collaboration to ensure equitable outcomes.

**Conclusion:**

AI holds immense promise in enhancing neurosurgical diagnostics, surgical planning, and postoperative care. Nonetheless, its responsible integration demands robust ethical and regulatory frameworks that prioritize patient safety, transparency, and equity. Interdisciplinary collaboration and continuous real-world validation remain essential to address ongoing clinical and ethical challenges as AI technologies evolve.

## Introduction

**Figure 1.**
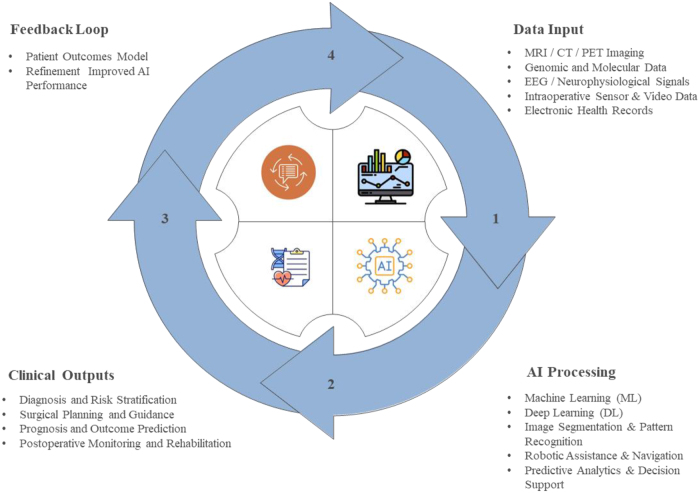
AI workflow in neurosurgery.

Artificial intelligence (AI) is rapidly transforming the healthcare landscape, and neurosurgery is no exception. By analyzing complex datasets and imaging findings, AI has enhanced diagnostic accuracy, optimized treatment planning, and improved surgical guidance, ultimately leading to better patient outcomes^[1]^. Advancements in AI algorithms have strengthened preoperative assessment and the interpretation of (Computed Tomography) CT, (Magnetic resonance imaging) MRI, and endoscopic images, facilitating intraoperative decision-making through machine learning, robotics, and deep data analysis^[2]^. Moreover, patient-specific anatomical models now allow highly detailed visualization of anatomical landmarks^[3]^. Despite the rapid evolution of AI-assisted neurosurgical technologies, comprehensive research on their ethical, legal, and societal implications remains limited. Given that neurosurgery involves high-risk and often irreversible procedures, an immediate and focused ethical discourse is essential for responsible AI integration. Understanding these issues is critical to ensuring that technological progress aligns with clinical best practices, equitable access, and patient safety. AI algorithms enable efficient tumor detection and classification by analyzing intraoperative images and performing radiomic, pathomic, and genomic analyses, thereby contributing to precise tumor profiling^[4]^. AI-based imaging techniques, particularly those employing deep learning on MRI and CT scans, facilitate early diagnosis and identification of strokes^[5]^. Additionally, AI supports clinicians in real time by identifying anatomical structures, tracking surgical tools, monitoring procedures, and predicting next steps during operations^[6]^. Innovations in robotic systems, magnified cameras, and minimally invasive surgical instruments have further enhanced visualization, precision, and accuracy^[7]^. Figure 1 illustrates the AI workflow in neurosurgery.

**HIGHLIGHTS**

- AI enhances neurosurgical diagnostics, planning, and postoperative care.
- Ethical concerns include bias, privacy, and accountability.
- Underrepresented datasets risk unequal patient outcomes.
- Over-reliance on AI may reduce human-centered care.
- Calls for transparent, ethical, and equitable AI integration.

### Revolutionizing diagnosis and management

AI is subtly revolutionizing neurosurgery by enhancing imaging techniques, such as stimulated Raman histology, which enables rapid intraoperative identification of tumor margins^[8]^. Through instantaneous assessment, AI can efficiently diagnose brain tumors, strokes, and aneurysms, surpassing traditional diagnostic methods^[9]^. In recent years, AI has gained considerable attention, driving major advancements across healthcare. These technologies hold transformative potential for patient data management, diagnostic accuracy, and personalized treatment planning, particularly in the medical field^[10]^. In neurology, AI is reshaping the prediction and diagnosis of neurological diseases by identifying intricate patterns that may escape human observation. Its role in continuous, real-time monitoring of parameters such as intracranial pressure and seizure activity has been further strengthened through wearable and implantable neuro-monitoring devices, enhancing perioperative care^[11]^. To facilitate early diagnosis of neurosurgical disorders such as brain tumors, aneurysms, and traumatic brain injuries, machine learning algorithms are increasingly being used to analyze brain imaging, especially MRI and CT scans, with high precision. AI’s application also extends to interpreting electroencephalograms (EEGs) and assessing cerebral artery blockages through fractional flow reserve CT, thereby reducing the likelihood of unnecessary interventions^[12]^. This narrative review distinguishes neurosurgical applications from general surgical AI use, focusing on issues such as bias, privacy, accountability, and the integration of AI into high-stakes operative decision-making.

### Ethical concerns in AI-powered neurosurgery

AI tools, particularly those used in neurosurgery, are only as effective as trained datasets. Inaccuracies and biases can occur where there is a lack of diversity, creating disparities in outcomes for minority and ethnic populations^[13]^. For instance, AI learning from a male-dominant dataset may introduce biases against female patients. This lack of diversity in race, age, sex, and geography may lead to variation in outcomes. Some algorithms might overestimate risks in specific populations, leading to unnecessary interventions or neglect^[14]^. Secondly, a high-risk concern is the sensitive nature of neurological information. We can collect intricate data, such as cognitive function, mental stability, and emotional patterns, through EEGs, brain-computer interfaces, and neuroimaging techniques. Such data is highly sensitive to misuse^[15]^. Thirdly, neurosurgical decisions are a high-risk affair; an error may have grave, severe consequences such as permanent disability or death. Here, the accountability of AI for causing such errors raises significant legal and ethical concerns. There are questions about whether we should hold doctors accountable for systemic flaws or if hospitals should be responsible for integrating and maintaining AI tools^[16]^. Lastly, one of the most significant concerns regarding AI in healthcare is the potential for dehumanization. The introduction of AI technologies may lead to over-reliance on machine-generated data, reducing the emphasis on human aspects of care. In neurosurgery, a field often requiring nuanced clinical judgment and patient-centered care, over-reliance on AI could undermine the patient-clinician relationship. While powerful, AI cannot replicate the empathy, intuition, and understanding human clinicians bring to patient care. It is important to strike a balance between AI-driven insights and considering the importance of human touch, ensuring that the healthcare system remains patient-centered and compassionate^[17]^.

**Figure 2.**
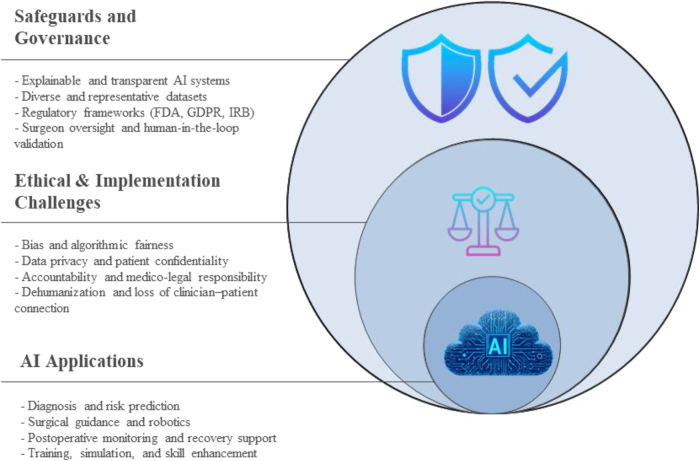
Conceptual framework diagram of ethical and implementation challenges of AI in neurosurgery.

Figure 2 depicts the conceptual diagram of the ethical and implementation challenges of AI in neurosurgery.

In this narrative review, we critically examine the ethical challenges posed by AI technologies in neurosurgery, focusing on issues of bias, privacy, accountability, and regulation, and how these factors influence clinical practice. Unlike broader AI applications in healthcare, neurosurgery presents unique ethical dilemmas due to its dependence on diverse datasets, real-time data collection from wearable devices, and the integration of AI into high-stakes decision-making processes. The review also highlights the need to balance AI advancements with human judgment to prevent overreliance on technology and the dehumanization of patient care^[18]^. Although AI in neurosurgery offers unprecedented capabilities, its adoption introduces specific ethical concerns, including bias from non-representative datasets, privacy risks associated with neurological data, accountability for AI-related errors, and fears surrounding the loss of human connection in care delivery. Given the critical nature of neurosurgical interventions, addressing these challenges is essential. This review explores these issues in depth and proposes strategies to support the ethical and equitable application of AI in neurosurgery.

This manuscript is made compliant with the TITAN checklist to ensure transparency in the reporting of AI ^[19]^.

## Methodology

This narrative review follows standard guidelines for synthesizing biomedical literature. A structured search was conducted using PubMed, the Cochrane Library, and Google Scholar to identify peer-reviewed articles published between January 2000 and March 2024. Search terms included combinations of “artificial intelligence,” “AI,” “machine learning,” “deep learning,” “neurosurgery,” “robotic surgery,” “ethics,” “bias,” and “data privacy.” The inclusion criteria for this review were as follows: peer-reviewed articles focused on the application of AI in neurosurgery, studies addressing the ethical, legal, and social implications of AI within this field, and articles published in English.

## Critical insights

### Current applications of AI in neurosurgery

**Figure 3.**
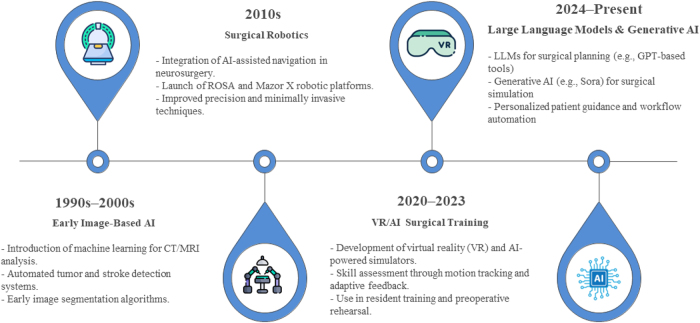
Timeline of AI in neurosurgery.

AI-driven image analysis tools have markedly advanced neurosurgical diagnostics, particularly in brain tumor classification and stroke assessment. For instance, deep learning models, such as the convolutional neural network developed bySiddique MA *et al* have demonstrated over 96% accuracy in distinguishing tumor types from MRI data^[20]^. AI-assisted surgical planning now enables the generation of patient-specific 3D visualizations of tumors, vasculature, and eloquent brain regions, supporting more precise approaches in procedures like aneurysm clipping and tumor resection^[21]^. Recent studies also highlight the value of AI-enhanced risk prediction models in guiding clinical decision-making, such as forecasting aneurysm rupture risk^[21]^. Neurosurgical robotic systems, including ROSA and Mazor X, integrated with AI algorithms, provide real-time intraoperative assistance, improving trajectory planning and targeting precision. Clinical trials have confirmed their safety and efficacy, especially in stereotactic and spinal surgeries^[21]^. Postoperatively, AI models are increasingly used to predict complications such as hemorrhage, infection, and hydrocephalus, while large-scale studies employing multitask deep learning on extensive patient datasets further enable early detection and timely intervention for in-hospital complications^[22]^. Figure 3 illustrates the timeline of AI in neurosurgery.

### Ethical considerations

Current ethical challenges in AI neurosurgery revolve around ensuring that patients fully understand the capabilities and limitations of AI tools, especially given the often opaque “black-box” nature of these systems^[23,24]^. Data privacy remains a critical issue due to the vast amounts of clinical and neural data required by AI. Compliance with regulations such as HIPAA and GDPR continues to evolve under the oversight of agencies like the FTC^[25]^. Bias in AI algorithms, often resulting from the underrepresentation of diverse populations in training datasets, poses a risk of unequal outcomes. Recent findings highlight the importance of developing inclusive datasets to help reduce such disparities^[23,24]^. Ongoing efforts toward explainable AI (XAI) aim to enhance transparency and build clinician trust by offering interpretable insights into model decisions^[23,24]^. Determining legal liability when AI-assisted interventions result in adverse outcomes remains complex, requiring careful consideration of responsibility among developers, clinicians, and institutions^[23,26]^. Another ethical concern involves potential overreliance on AI, which could erode neurosurgeons’ clinical judgment and skills. Therefore, AI should function as an augmentation tool rather than a replacement, preserving the primacy of human expertise in surgical training and decision-making^[24,26]^.

Access to advanced AI technologies is often limited to well-resourced healthcare settings, raising concerns about widening existing healthcare disparities^[23]^. Addressing this issue requires coordinated efforts to ensure the equitable distribution and implementation of AI tools across diverse clinical environments^[23,24]^. Given the rapid pace of AI development, ongoing multidisciplinary collaboration among ethicists, clinicians, data scientists, and policymakers is essential to establish nuanced ethical guidelines that reflect the evolving landscape of AI integration in neurosurgery^[23,24,26]^. Concrete and actionable recommendations should be tailored to the needs of neurosurgeons, hospital administrators, and regulatory bodies to promote responsible and effective adoption^[23,26]^. Although large language models such as ChatGPT show growing potential in medical education and communication, their direct application in neurosurgical practice remains under evaluation. Future research should clarify and rigorously assess the use of such technologies within neurosurgical contexts to prevent overstating their current clinical relevance^[27,28]^.

In cerebrovascular care, AI platforms such as Viz.ai and RapidAI now analyze CT and MRI scans to detect acute ischemic strokes and hemorrhages with high sensitivity, enabling faster clinical interventions^[29,30]^. More recent algorithms have advanced even further; for instance, a YOLOv7-based model achieved approximately 99.5% accuracy in detecting and localizing common brain tumors on MRI scans^[31]^. These predictive models are also being applied to forecast long-term outcomes. Machine learning has demonstrated strong performance in predicting tumor recurrence and functional recovery following surgery, offering valuable insights for rehabilitation planning^[32]^. Beyond diagnostics and prognostication, AI is transforming neurosurgical training and education. Virtual reality (VR) simulators enhanced with AI analytics allow surgeons to rehearse complex procedures in realistic, risk-free environments while receiving personalized performance feedback. Notably, one study found that trainees who received AI-augmented, instructor-guided feedback significantly outperformed their peers in simulated neurosurgical tasks^[33]^. Collectively, such findings suggest that AI-enhanced training accelerates skill acquisition and supports, rather than replaces, human expertise in surgical education^[33]^.

Experts emphasize that promoting equity is crucial: recent reviews call for developing open-access AI tools and infrastructure to avoid widening the neurosurgical AI care gap^[21,34]^. Notably, scholars have urged comprehensive, multidisciplinary governance: a recent systematic review highlighted the need for transparent AI use, regulatory oversight, and up-to-date ethical guidelines to ensure safe, equitable integration of AI in neurosurgery^[24,34]^. Table 1 summarizes selected studies and their key findings.

**Table 1:** Selected studies with key findings

Author	Key findings
Ryvlin *et al*^[35]^	AI, ML, and intraoperative imaging enable personalized care, tumor detection, and minimally invasive approaches. Balancing innovation with ethical responsibility is critical.
Awuah *et al*^[21]^	ML/DL models improve diagnostics, surgical planning, and prognosis prediction (e.g., gliomas, metastases) but require prospective validation.
Titov *et al*^[36]^	ML algorithms (SVM, k-NN) classify expertise via VR/surgical videos with >90% accuracy, outperforming human raters in skill evaluation.
Windermere *et al*^[37]^	AI enhances training and reduces complications, but it underperforms residents in tests; retrospective studies dominate, and real-world trials are needed.
Zhang *et al*^[38]^	Text-to-video AI (Sora) aids surgical planning, immersive training, and patient communication, but raises ethical/access concerns.
Tangsrivimol *et al*^[39]^	ML achieves high accuracy in glioma/metastasis segmentation, prognosis, and intraoperative guidance, thereby optimizing precision in tumor surgery.
Bravo *et al*^[40]^	AI/robotics improve stroke/aneurysm diagnostics and catheter stability, reduce radiation, and enhance endovascular procedural efficiency.

AI, Artificial intelligence, DL, Deep learning, ML, Machine Learning, VR, Virtual Reality

**Figure 4.**
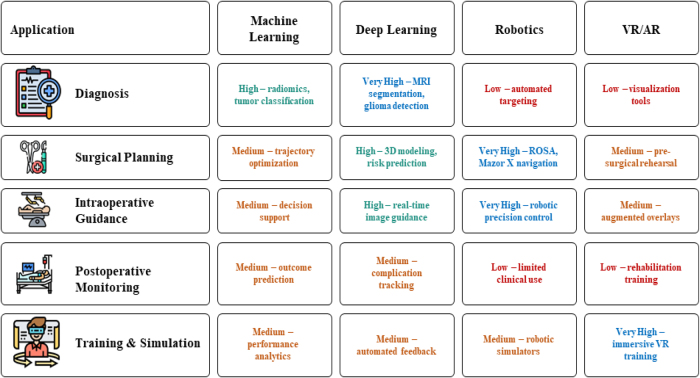
Relationship between clinical applications and AI methods in neurosurgery.

Figure 4 showcases the relationship between clinical applications and AI methods in neurosurgery.

### Challenges in implementation

AI holds enormous promise for enhancing both clinical and administrative healthcare tasks; however, its adoption has been notably slow, primarily due to significant implementation challenges. A major barrier lies in the insufficient consideration of the broader sociotechnical systems within which AI operates. Poor integration with existing clinical workflows and organizational structures often leads to limited user acceptance, increased error risk, patient safety concerns, and higher long-term costs, thereby undermining anticipated efficiency gains. The uptake of AI is further constrained by inadequate algorithm validation, poor data quality, and limited external replication. Many AI models remain underutilized by clinicians due to concerns about scalability and generalizability. Moreover, the legal and regulatory frameworks governing AI in healthcare remain underdeveloped. The lack of representation of diverse racial and ethnic groups in training datasets also poses a risk of perpetuating existing biases and mirroring disparities seen in other areas, such as pharmaceutical development. While AI holds considerable promise for advancing neurosurgical practice, its current role remains largely supportive, most commonly applied to image segmentation, diagnostic assistance, and data analysis rather than replacing core clinical decision-making. Nevertheless, some experts have expressed concern that excessive long-term reliance on AI could gradually erode hands-on surgical skills and critical thinking abilities if adequate safeguards are not established^[39,41,42]^.

Technical vulnerabilities, such as software errors or hardware malfunctions, though relatively uncommon, underscore the need for robust maintenance and contingency planning to ensure continuous and reliable system performance. Equally important is transparency in AI decision-making; clinicians must understand how outputs are generated to verify their accuracy and assess their clinical relevance. Preserving clinical judgment, accountability, and physician oversight remains essential to maintaining patient safety in neurosurgical care. As automation capabilities expand, these human-centered principles may face increasingly complex challenges, particularly in defining legal responsibility when AI influences clinical decisions. The rise of generative AI further introduces ethical and data security concerns, especially when used for sensitive tasks such as drafting clinical documents or managing medical records. Although these challenges are not yet prevalent in neurosurgical workflows, proactively addressing them now will help ensure responsible and balanced AI integration in the future^[39,41,42]^. The primary risks associated with medical AI arise from the design and function of its underlying algorithms. As AI becomes more deeply embedded in clinical decision-making, there is an urgent need for modernized regulatory frameworks that prioritize patient safety and uphold individual autonomy. A key concern is that AI could inadvertently override physicians’ clinical judgment, promoting standardized recommendations that fail to account for patients’ unique values and treatment preferences. For example, AI-generated outputs may emphasize statistical measures such as projected life expectancy, which may not align with personal goals like comfort or quality of life. Without appropriate safeguards, increasing reliance on AI risks eroding the doctor–patient relationship, reducing shared decision-making, and promoting a more impersonal, mechanized approach to care. Furthermore, the high initial costs associated with implementing AI systems in therapeutic settings present both financial and ethical challenges for healthcare institutions^[43]^.

Despite these concerns, successful examples of AI integration demonstrate its potential in enhancing diagnostic accuracy and surgical planning. A study published in Academic Radiology introduced a machine learning model based on radiomic analysis of multiparametric MRI scans, designed to stratify prognosis in pediatric patients with medulloblastoma. The model showed strong predictive performance, achieving an AUC of 0.946 in the training set and 0.797 in the validation set^[44]^.

### Strategies for addressing ethical challenges

The integration of AI into healthcare research represents a significant step forward, with the potential to enhance diagnostic precision, inform therapeutic planning, and improve overall patient management. Yet, this rapid evolution also brings complex ethical challenges that require deliberate, context-specific solutions. In neurosurgery, where decisions often carry life-altering consequences, the stakes are even higher. A primary concern is the safeguarding of patient privacy and data confidentiality, given that AI systems rely on extensive datasets that may expose individuals to security breaches, unauthorized access, or misuse^[45]^. A more pragmatic approach involves role-specific actions for neurosurgeons, hospital administrators and regulatory bodies directly engaged in AI deployment.

**Neurosurgeons** should verify AI performance using their institution’s patient datasets before clinical adoption to ensure relevance to the local demographic and case mix. Ongoing professional training on AI capabilities and limitations is essential, enabling surgeons to critically evaluate algorithm outputs and identify potential biases. Maintaining a structured record of cases in which AI influenced clinical decisions can further support retrospective review and quality improvement^[45]^. **Hospital administrators** can enhance AI governance by implementing tiered data access controls, robust encryption protocols, and clearly defined breach-response procedures. Dedicated funding should be allocated for independent audits to monitor algorithmic bias and detect performance degradation over time. Establishing an interdisciplinary AI oversight board comprising clinicians, data scientists, and patient representatives can provide an additional layer of ethical review before new systems are deployed^[45]^. **Regulatory bodies** also play a pivotal role in defining neurosurgery-specific standards for AI validation, including requirements for diverse and representative datasets and ongoing post-market surveillance of system performance. Furthermore, regulations should mandate minimum transparency standards to ensure that AI-generated recommendations remain explainable and open to clinician scrutiny. Regular certification and compliance checks would help uphold safety and accountability^[45]^.

## Future directions and recommendations

### Advancing predictive analytics: refining models for early detection of neurological conditions

AI has significantly improved the early detection of neurological disorders by analyzing complex neuroimaging data. Machine learning techniques play a crucial role in diagnosing and predicting cognitive neurodegenerative diseases, such as Alzheimer’s disease, through modalities like MRI and PET scans. These findings underscore AI’s potential to enhance diagnostic accuracy and enable earlier intervention strategies. Powered by AI, predictive models are transforming the early diagnosis of neurodegenerative illnesses. Borchert *et al* demonstrated that deep learning applied to MRI data could detect Alzheimer’s disease with over 90% accuracy in preclinical stages^[46]^. Similarly, Yousefi *et al* showed that predicting mild cognitive impairment using machine learning and multimodal biomarkers improved early therapeutic targeting^[47]^. Feng *et al* further highlighted AI’s role in detecting diseases such as Alzheimer’s and Parkinson’s by analyzing complex datasets, thereby improving early diagnosis and treatment planning^[48]^.

### Enhancing non-invasive diagnostics: AI for portable imaging and real-time feedback

AI has revolutionized non-invasive diagnostics in neurology by improving the interpretation of imaging data. Advanced imaging techniques, including diffusion tensor imaging (DTI) and magnetic resonance spectroscopy (MRS), when combined with AI algorithms, have enhanced the ability to distinguish tumor progression from treatment-related changes in brain tumors, allowing for more precise and less invasive evaluation^[49]^. In the context of autism spectrum disorder (ASD), AI models analyzing functional MRI (fMRI) data have demonstrated the capacity to identify distinct neural activation patterns associated with ASD, contributing to early detection and intervention^[50]^. Furthermore, integrating AI with liquid biopsy techniques offers a non-invasive method for diagnosing and monitoring brain tumors. By detecting tumor-derived biomarkers in bodily fluids, AI-enhanced liquid biopsies provide real-time insights into tumor dynamics, supporting personalized treatment strategies^[51]^.

### AI-enhanced wearable devices: improving monitoring of neurological parameters

Recent advances in AI-integrated wearable devices have enabled continuous monitoring of neurological parameters in real-world environments. For instance, a portable physiograph has been utilized to assess gait patterns in patients with Parkinson’s disease (PD)^[52]^. Convolutional neural networks were applied to biomechanical data to distinguish between physiological and PD-specific walking, offering a non-invasive method for disease monitoring and management^[52]^.

### Addressing ethical and privacy concerns: developing bias-mitigation tools, explainable AI, and privacy frameworks

Integrating AI into neurological diagnosis raises significant ethical and privacy challenges. Algorithmic bias can lead to disparities in health outcomes, emphasizing the need for fairness and inclusivity in AI design. Kocak *et al* highlighted the importance of identifying and mitigating biases in medical imaging algorithms to prevent adverse impacts on patient care^[53]^. Moreover, the lack of transparency in AI decision-making, often referred to as the “black-box” problem, poses clinical concerns. Implementing XAI techniques can enhance interpretability and foster trust between clinicians and AI systems^[54]^. Privacy concerns also emerge from the processing of sensitive patient data, underscoring the need for strong data protection measures and adherence to ethical standards for responsible AI use. Benjamin et al. emphasized the role of regulatory bodies such as the U.S. Food and Drug Administration (FDA) and the European Medicines Agency (EMA) in overseeing AI and machine learning models. Before any medical hardware or software is approved for the U.S. market, developers must undergo FDA evaluation, which involves three levels of regulatory clearance for medically oriented AI/ML-based algorithms^[55]^.

**Figure 5.**
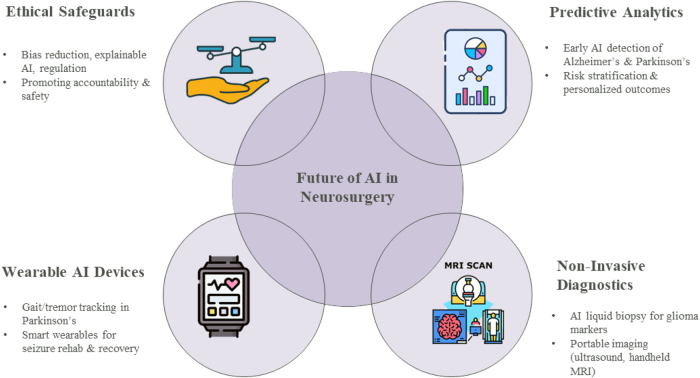
Radial diagram of the future of AI in neurosurgery.

Figure 5 depicts the radial diagram of the future of AI in neurosurgery.

### Gaps

#### Understanding sources of bias in AI datasets

Representation bias in AI datasets may arise from various factors, including historical discrimination, selection bias, and sampling errors during data acquisition and preparation. It is unrealistic to expect AI-based solutions to produce equitable outcomes without addressing representation bias at the dataset level. While fairness in machine learning models has been widely studied, bias within the data itself has received comparatively less attention. This paper reviews existing literature on identifying and addressing representation bias as a property of datasets, independent of how they are later applied^[56]^. Tripathi *et al* analyzed publicly available medical imaging datasets and concluded that underrepresentation of certain demographic groups can introduce systemic bias in AI models^[57]^. Similarly, Stanley *et al* proposed a framework for systematically evaluating how biases in medical images affect AI model performance^[58]^. By detecting and quantifying these biases, researchers can develop strategies to ensure fairness and generalizability in AI applications.

#### Assessing bias impact on clinical outcomes

The presence of bias in AI models can significantly affect the accuracy of clinical decision-making. In cardiovascular imaging, for example, biased algorithms have been shown to negatively influence patient outcomes, underscoring the importance of rigorous validation before clinical deployment^[59]^. Moreover, biases in medical AI can perpetuate existing healthcare disparities^[60]^. Addressing these biases is critical to ensuring equitable healthcare delivery and preventing the exacerbation of systemic inequalities.

#### Prioritizing explainability and interpretability of AI systems

Implementing AI in neurology requires models that are both accurate and transparent. XAI techniques are being developed to clarify the decision-making processes underlying AI models. For example, Vision Transformation (VT) technologies propose self-explanatory alternatives to traditional convolutional neural networks, enhancing transparency and interpretability in diagnostic applications^[49]^.

## Conclusion

AI is transforming neurosurgery by analyzing vast datasets to support diagnosis, surgical planning, and the management of complex neurological conditions. However, its rapid integration also introduces ethical, technical, and societal challenges that require urgent attention. Data vulnerabilities, algorithmic bias, and opaque “black-box” systems pose substantial risks to patient privacy and autonomy. To mitigate these concerns, transparent and interpretable frameworks must be developed. Regulatory bodies should establish adaptive standards for AI validation and mandate rigorous real-world testing to address technical limitations and software vulnerabilities that hinder seamless clinical adoption. Effective human–AI collaboration is essential to enhance precision, expand accessibility, and improve overall care standards. Interdisciplinary partnerships are equally vital for developing equitable AI solutions that prioritize patient safety and inclusivity. Researchers are now advancing portable MRI analyzers powered by interpretable AI, yet their ethical deployment demands strong governance and international collaboration. Ultimately, the convergence of human compassion and machine intelligence holds the promise of a new era in neurosurgery, one where technology genuinely serves humanity, ensuring that every patient receives exceptional and equitable care.

## Data Availability

All data used in this narrative review are publicly available and sourced from previously published studies. No new data were generated for this work. All included articles have been appropriately cited within the manuscript and are available through the references section.

## Research registration unique identification number (UIN)

The Research registration unique identification number (UIN) was not required because it is a narrative review.

## Provenance and peer statement

Not commissioned, externally peer-reviewed.
